# Fabrication of *Helix aspersa* Extract Loaded Gradient Scaffold with an Integrated Architecture for Osteochondral Tissue Regeneration: Morphology, Structure, and *In Vitro* Bioactivity

**DOI:** 10.1021/acsabm.2c01050

**Published:** 2023-04-03

**Authors:** Sedef Tamburaci, Merve Perpelek, Selma Aydemir, Basak Baykara, Hasan Havitcioglu, Funda Tihminlioglu

**Affiliations:** † Department of Chemical Engineering, İzmir Institute of Technology, Urla, Izmir 35430, Turkey; ‡ Department of Biomechanics, Dokuz Eylul University, İzmir 35330, Turkey; § Department of Histology and Embryology, Dokuz Eylul University, İzmir 35330, Turkey; ∥ Department of Orthopedics and Traumatology, Dokuz Eylul University, İzmir 35330, Turkey

**Keywords:** *Helix aspersa*, gradient scaffold, osteochondral, mucus, slime

## Abstract

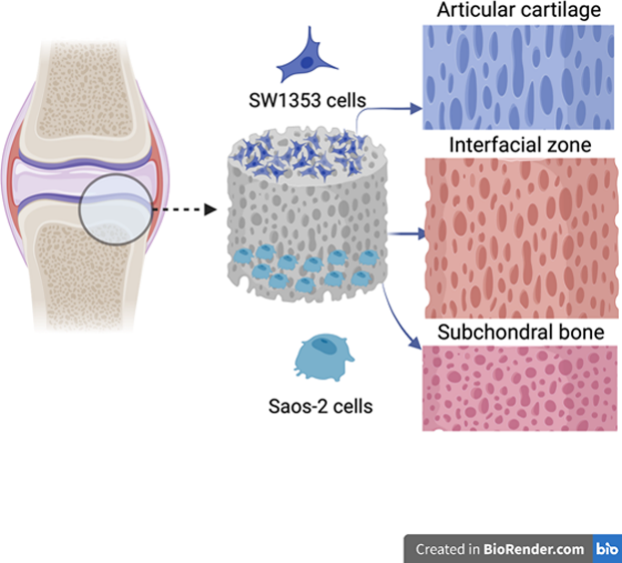

Regeneration of osteochondral tissue with its layered complex structure and limited self-repair capacity has come into prominence as an application area for biomaterial design. Thus, literature studies have aimed to design multilayered scaffolds using natural polymers to mimic its unique structure. In this study, fabricated scaffolds are composed of transition layers both chemically and morphologically to mimic the gradient structure of osteochondral tissue. The aim of this study is to produce gradient chitosan (CHI) scaffolds with bioactive snail (*Helix aspersa*) mucus (M) and slime (S) extract and investigate the structures regarding their physicochemical, mechanical, and morphological characteristics as well as *in vitro* cytocompatibility and bioactivity. Gradient scaffolds (CHI-M and CHI-S) were fabricated via a layer-by-layer freezing and lyophilization technique. Highly porous and continuous 3D structures were obtained and observed with SEM analysis. In addition, scaffolds were physically characterized with water uptake test, micro-CT, mechanical analysis (compression tests), and XRD analysis. *In vitro* bioactivity of scaffolds was investigated by co-culturing Saos-2 and SW1353 cells on each compartment of gradient scaffolds. Osteogenic activity of Saos-2 cells on extract loaded gradient scaffolds was investigated in terms of ALP secretion, osteocalcin (OC) production, and biomineralization. Chondrogenic bioactivity of SW1353 cells was investigated regarding COMP and GAG production and observed with Alcian Blue staining. Both mucus and slime incorporation in the chitosan matrix increased the osteogenic differentiation of Saos-2 and SW1353 cells in comparison to the pristine matrix. In addition, histological and immunohistological staining was performed to investigate ECM formation on gradient scaffolds. Both characterization and *in vitro* bioactivity results indicated that CHI-M and CHI-S scaffolds show potential for osteochondral tissue regeneration, mimicking the structure as well as enhancing physical characteristics and bioactivity.

## Introduction

The osteochondral tissue has a unique multilayered and complex structure composed of the articular cartilage, cartilage–bone interface, and subchondral bone with different architectures at each layer. Thus, regeneration of osteochondral tissue defects requires more sophisticated scaffold designs. Degenerative changes result from osteochondral defects that arise from the damage of both the full-thickness articular cartilage and the underlying subchondral bone zone that leads to the mechanical instability of the joint.^1,2^ The articular cartilage consists of chondrocytes and extracellular matrix (ECM) components such as water, collagen fibers, and proteoglycans. It has structurally four layers (superficial, middle, deep, and calcified cartilage) with unique properties.^3,4^ While the superficial region indicated strong resistance to shear forces, as the depth increases, the matrix protein network originated different types of collagen (types II, IX, XI, and VI) and has a composition that can resist compression stress.^5,6^ The subchondral bone provides biomechanical support to the articular cartilage and exists below the calcified zone in the articular cartilage. Load transfer between the articular cartilage and subchondral bone is provided by the bone–cartilage interface. Because the bone–cartilage interface has a complex structure, the technical term “tidemark” is used to separate the two structures.^7^ As the cartilages with a structure having lack of vessel, nerve, and lymph tissues, regeneration of osteochondral tissue is quite difficult itself after injury or degenerative diseases.^8^ The subchondral bone and cartilage tissue have an interconnected functional structure, and as cartilage lesions progress, the subchondral bone is damaged. Studies demonstrated that the subchondral bone should be treated at the same time to repair the cartilage injury.^9−11^ If the damaged area of the tissue is small, osteochondral lesions are repaired by the formation of a deficient functional fibrocartilaginous tissue derived from mesenchymal stem cells of the bone marrow, but if the damaged area is larger, the cartilage tissue cannot repair and regenerate itself.^12^ Clinical treatments are available for osteochondral injuries such as arthroplasty, subchondral drilling, microcracking, prosthetic joint replacement, and allograft–autograft applications.^9,13,14^ However, these methods have many disadvantages such as physiological skin and tissue damage and biological complications at surgical procedure such as infection, tissue incompatibility, and immunological reactions.^15−18^ In recent years, many scaffold designs have been fabricated using different techniques for osteochondral tissue regeneration. Initially, monolayer scaffolds were produced, but then, bilayer and multilayer scaffolds have been fabricated to mimic the natural morphology of the osteochondral tissue by using biopolymers, cells, and growth factors.^4,10,19−26^ The articular cartilage and subchondral bone zones have different morphologies leading to distinct structural characteristics and physiological functions. Thus, conventional single-layer biomaterials with homogeneous properties may not be appropriate to provide the ideal microenvironment for osteochondral regeneration.^2^ Therefore, osteochondral defects in the cartilage and subchondral bone zones can be supported by integrated multilayered materials with unique microstructures. The morphological and biological characteristics of natural tissues, which are composed of interfacial zones between tissues, cannot be replicated by these multilayered scaffold designs despite their enormous promise for mending osteochondral lesions because an interface zone forms during the integration of these two distinct tissue layers, which are made up of various cell types. Recent research has concentrated on discrete gradient multilayer scaffold designs to overcome these constraints as well as to smoothly build the transition zones from hard tissue to soft tissue to accurately imitate anatomical characteristics.^27−31^ There are, however, few investigations on scaffold designs as continuous gradient layered structures for regenerating osteochondral tissue.

The use of natural polymers in biomaterial design for hard tissue regeneration leads to weak physical characteristics especially in terms of mechanical properties. Thus, bioactive agents have been recently used as reinforcement agents in biopolymer matrices to mimic the composite structure of hard tissue as well as cope with their limitations. Among bioactive sources, garden snail *Helix aspersa* secretions have come into prominence with their rich biologically active components such as allantoin; collagen; elastin; glycolic acid; GAG; and vitamins A, E, and C. *H. aspersa* secretions are generally classified as mucus and slime; mucus is produced for adhesive properties, and slime is produced for moving that snails leave behind during their movement. Literature studies indicated the effects of snail secretion by inducing fibroblast proliferation and enhancing wound healing.^32,33^

In our previous study, we fabricated mucus and slime extract loaded single-layer porous chitosan scaffolds for bone and cartilage tissue regeneration separately. We investigated the effects of different mucus and slime extract loading percentages on each scaffold in terms of physical characteristics and bioactivity. CHI-M and CHI-S scaffolds were obtained with enhanced mechanical properties and high biodegradation rates, whereas mucus and slime extract loading in chitosan matrix induced *in vitro* bioactivity in terms of ALP activity, biomineralization, and GAG formation for bone and cartilage tissue regeneration.^34^ However, bone–cartilage interfaces require integrated transition layers and morphological alterations where each different layer and transition zone should meet the needs of bone–cartilage tissue layers with regard to morphological characteristics and bioactivity. Thus, the aim of this study is to develop an alternative morphologically gradient layered scaffold with transition layers capable of mimicking osteochondral tissue morphology continuously for bone, cartilage, and joint injuries as well as enhancing the bioactivity by incorporation of mucus and slime extracts with gradient concentrations to obtain bioactive zones for both bone and cartilage regeneration.

## Experimental Section

### Materials

Medium-molecular-weight chitosan powder (Sigma-Aldrich), *Helix aspersa* mucus extract (Medical Grade, Xi’an SR Bio-Engineering Co., Ltd.), and *Helix aspersa* slime extract (Pharmaceutical Grade) (Xi’an Nate Biological Technology Co., Ltd.) were used for scaffold fabrication. Eagle’s MEM (Capricorn Scientific), penicillin/streptomycin, and l-glutamine (Lonza) were used in cell culture studies. The WST-1 assay (BioVision Inc.), Enzyline PAL Optimise ALP kit (Biomerieux, France), Human Osteocalcin and Human Cartilage Oligomeric Matrix Protein (COMP) Sandwich-ELISA Kits (Elabscience), and proteoglycan assay (Amsbio, AMS Biotechnology) were used for *in vitro* bioactivity studies. Hematoxylin & eosin (Mayer’s), Masson’s Trichrome (Bio-Optica, 04-010802), Alcian Blue (ScienCell 8378), Alizarin Red S Staining kit (Abcam ab146374), Safranin O Staining kit (ScienCell 8348), type I collagen (bs-10423R, Bioss), and type II collagen (bs-0709R, Bioss) were used for histology staining protocols. Silver nitrate and sodium thiosulfate (Sigma-Aldrich) were used for the biomineralization study.

### Fabrication of CHI-M and CHI-S Gradient Scaffolds

**Scheme 1:**
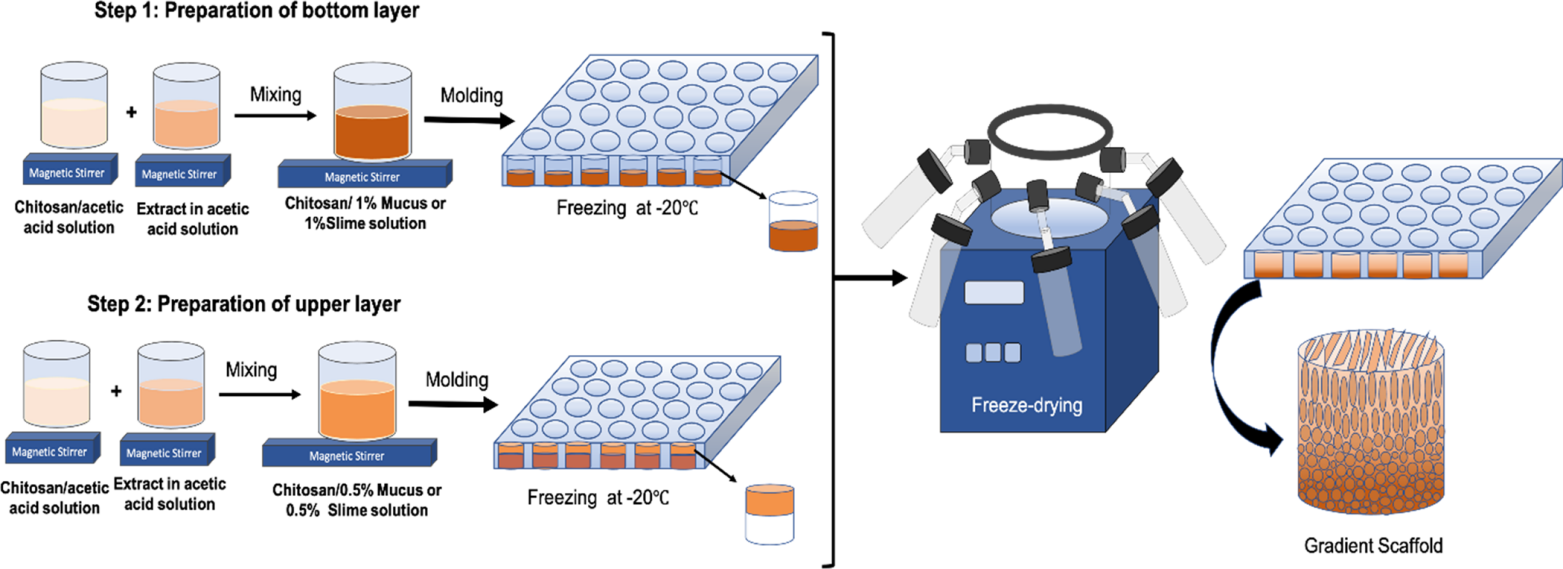
Schematic View of the Fabrication Process of the Gradient Scaffold Preparation of upper and bottom layer solutions, layer-by-layer freezing, and freeze-drying steps.

Gradient scaffolds were prepared with layer-by-layer prefreezing and fabricated with the freeze-drying method as shown in Scheme 1 in detail. Each layer was prepared with different mucus or slime loading percentages in the chitosan solution and then molded and prefrozen one on the top of another. Mucus loaded chitosan (CHI-M) and slime loaded chitosan (CHI-S) solutions were prepared by dissolving chitosan (1% wt) in acetic acid solution (1% v/v) and mucus or slime powder (0.5% wt for the upper layer and 1% wt for the bottom layer) dispersed in acetic acid solution (1% v/v) separately. Then, the chitosan solution and mucus or slime dispersion were blended in magnetic a stirrer overnight. The bottom layer 1% wt CHI-M and 1% wt CHI-S solution was poured into 48-well plates and prefrozen at −20 °C for 24 h (Scheme 1, step 1). Then, the upper layer 0.5% wt CHI-M and 0.5% wt CHI-S solution was poured on the frozen solution and frozen at −20 °C for 24 h again (Scheme 1, step 2). Samples were lyophilized at −46 °C and 0.018 mbar vacuum for 48 h to fabricate the gradient layer structure. By this way, two different prefrozen solutions constituted laminated continuous gradient layers in the scaffold structure (Scheme 1, gradient scaffold).

### Scanning Electron Microscopy (SEM) Analysis

Morphological characterization of the obtained gradient scaffolds was performed by scanning electron microscopy (Quanta FEG). Scaffolds were coated with gold under argon gas before the analysis. The average pore size of each layer was determined with the ImageJ software.

### Water Absorption Capacity of Scaffolds

The dry weight (*W*_0_) of the samples was determined before the absorption study. Then, the scaffolds were neutralized with 1 M NaOH solution for 30 min and washed twice with PBS solution (1×) to remove excess NaOH. After the washing step, samples were incubated in PBS solution (1×) at 37 °C for 4, 24, and 48 h. Before measurement, excess water was removed with a filter paper, and samples were weighed (*W*_1_). The water absorption capacity percentage of the scaffolds was calculated using the following equation:
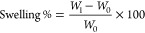
1

### Compression Test

Mechanical analyses of scaffolds (*n* = 5) were performed with a compression test according to the ASTM D 5024-95a standard (TA-XT Plus Texture Analyzer). The device was set to apply a 5 kN load and 5 mm/min speed. Compression was applied to 75% strain of each sample. Compression test measurements were given as elastic modulus.

### Micro-CT Analysis

The porosity and 3D microstructure of gradient scaffolds were investigated by microtomography (Scanco-μCT 50). Samples were scanned with penetrative X-rays of 45 kVp and 88 μA at a native resolution using an air filter. Each specimen was visualized using 500 slices, and the 3D structure was investigated with a 3 μm voxel size.

### Exogenous Mineralization Study

Exogenous mineralization on CHI-M and CHI-S gradient scaffolds was carried out for 14 days by incubating in 10× modified simulated body fluid solution (SBF) at 37 °C to mimic the *in vivo* microenvironment. The SBF medium was refreshed every 48 h to mimic the circulation and prevent the decrease of free calcium and phosphate ions during incubation. Hydroxyapatite formation on the scaffold surface was investigated with XRD analysis (Philips X’PertPro MRD). Diffraction patterns of mineral deposition on samples were investigated at 40 kV using Cu Kα radiation (λ = 1.54 Å). Peak intensities were recorded between 10 and 80° (2θ) with a 0.139° s^–1^ scanning rate.

### *In Vitro* Studies

Human osteosarcoma (Saos-2) and chondrosarcoma (SW1353) cell lines were used as model cells for *in vitro* studies. Eagle’s MEM (10% FBS, 1% l-glutamine, and 1% penicillin–streptomycin) was used for cell cultivation for 28 days. The osteogenic culture medium for Saos-2 cultivation was prepared with Eagle’s MEM supplemented with 1% beta-glycerophosphate and 0.1% ascorbic acid to induce secretion of osteogenic biomarkers.

### Cytotoxicity Tests

The *in vitro* cytotoxicity of mucus and slime loaded gradient scaffolds was evaluated with the indirect extraction method (ISO-10993 standard). Sample extracts were obtained by incubating scaffolds in MEM for 24 h at 37 °C. Extraction media of samples were used as culture media for cultivation. Cells only cultured with MEM were used as the negative control group. The WST-1 assay was used to measure the cell viability (440 nm) for 24, 48, and 72 h periods. Absorbance data were normalized to the cell viability according to the equation of the cell viability percentage as follows:
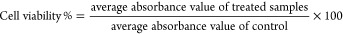
2

### Cell Proliferation

Saos-2 and SW1353 cells were co-cultured on each layer of gradient scaffolds with the direct co-culture method allowing cell–cell interaction. Saos-2 and SW1353 cell seeding was carried out at the same time point on the upper and lower parts of gradient scaffolds, respectively. Cell proliferation on gradient scaffolds was investigated for 28 days. Scaffolds were incubated at 37 °C and 5% CO_2_ during cultivation. Before cultivation, scaffolds were sterilized by incubating in 70% (v/v) ethanol solution overnight. After sterilization, scaffolds were washed with PBS solution (1×) to remove residual ethanol solution. Before cell seeding, scaffolds were conditioned with culture media for 2 h. Saos-2 and SW1353 cells were counted and seeded at a density of 2 × 10^6^ cell/mL. After cell seeding, samples were incubated for 4 h at 37 °C for initial cell attachment on the scaffold surface. The cultivation medium was refreshed twice a week. The WST-1 assay was used to detect cell viability at 440 nm (Varioskan Flash, ThermoFisher Scientific).

### Cell Attachment and Spreading on Gradient Scaffolds

Saos-2 and SW1353 cells were seeded simultaneously on each layer of gradient scaffolds and co-cultured with the direct method for 7 days to observe attachment and spreading. After cultivation, cell fixation was carried out with 4% paraformaldehyde solution (PFA) (v/v) for 20 min at room temperature. Then, samples were washed with PBS solution (1×) and dehydrated with ethanol solution in graded series (50, 70, 80, 90, and 100%) before SEM imaging.

### Alkaline Phosphatase Activity (ALP) and Osteocalcin (OC) Secretion

Saos-2 cells were cultivated on gradient scaffolds with the osteogenic medium for ALP and OC detection. ALP secretion of Saos-2 cells was measured spectrophotometrically at 7, 14, and 21 days of incubation. Osteocalcin production was measured with an ELISA assay at 21 and 28 days.

### Determination of Biomineralization with von Kossa Staining

Saos-2 cells cultivated on porous layer gradient scaffolds were fixed, stained, and observed at the 28th day for biomineralization. The cell fixation protocol was given in the Cell Attachment and Spreading on Scaffolds section previously. After fixation, samples were washed thrice using distilled water to remove PFA solution. Phosphate deposition on the porous layer of gradient scaffolds was carried out with the von Kossa staining protocol and given in our previous study.^34^ Biomineralization on scaffold surface was visualized with a stereomicroscope (Olympus SOIF DA 0737).

### Determination of the Cartilage Oligomeric Matrix Protein (COMP) and ECM Protein Glycosaminoglycans (GAGs)

The COMP activity of SW1353 cells was detected with an ELISA assay on the third and seventh days. GAG production was evaluated at the 14th, 21st, and 28th days with the spectrophotometric proteoglycan assay (530 nm). Before the proteoglycan assay, papain extraction was carried out to obtain GAG content. The papain extraction protocol was given in our previous study in detailed form.^34^

### Alcian Blue Staining

GAG secretion of SW1353 cells on gradient scaffolds was also determined with Alcian Blue staining at the 28th day of incubation. First, the scaffolds were washed twice with 1× PBS solution and fixed with 4% paraformaldehyde solution. Afterward, scaffolds were washed with distilled water and kept in the Alcian Blue solution (0.1%) in HCl solution (0.1 N) overnight at room temperature. The dye was removed by washing continuously with distilled water, and the GAG content was visualized with an optical microscope.^35^

### Histological and Immunohistochemical Analyses

The cell morphology, adhesion, and distribution as well as production of extracellular matrix components were evaluated with histochemical and immunohistochemical staining at 7, 14, 21, and 28 days of incubation.

### Histological Analysis

The sample tracking procedure was carried out with 4% paraformaldehyde fixation and paraffin embedding. Sections were obtained with 5 μm thickness and deparaffinized. Hematoxylin & eosin, Safranin O/Fast Green, Alcian Blue, Alizarin Red S, and Masson’s Trichrome stains were used for cell and ECM imaging.

### Immunohistochemical Staining

Immunohistochemical evaluation was carried out with 5 μm sections. After the dehydration stage, samples were treated with primary antibodies, type I collagen, and type II collagen. Then, treated samples were incubated at +4 °C overnight. The reaction was made visible with the diaminobenzidine (DAB) kit. Ground staining was carried out with Mayer’s hematoxylin staining. Imaging (Olympus BX51 microscope) was performed with 100× magnification. Semiquantitative scoring of immunoreactivity intensity was defined as no staining (0; −), low staining (1; +), moderate staining (2; ++), and severe staining (3; +++).^36,37^

### Statistical Analysis

Samples were used with three replications in characterization tests and *in vitro* studies. Five samples from each group were used for the mechanical experiment according to the ASTM standard. The experimental data were given with the standard error of the mean (SEM). Statistical analyses were carried out using one-way ANOVA and two-way ANOVA with Tukey’s multiple comparison test statistical methods (*p* < 0.05).

## Results and Discussion

### Morphology and Microstructure of Gradient Scaffolds

**Figure 1:**
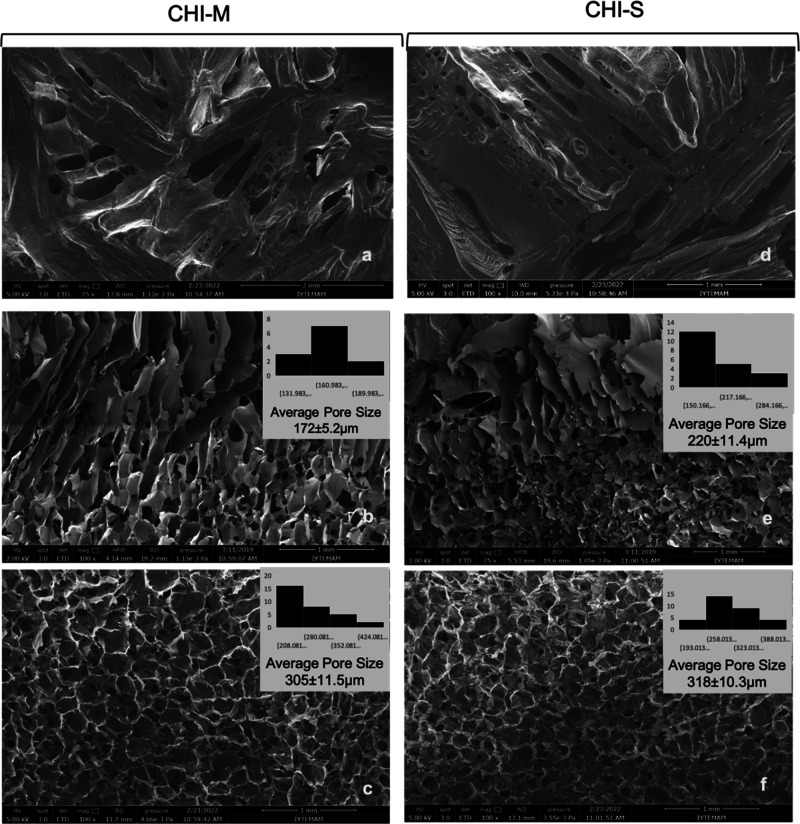
SEM images of upper, transient, and bottom layers of CHI-M (a–c) and CHI-S (d–f) gradient scaffolds with 75× and 100× magnifications. Average pore sizes of transient and porous bottom layers were given with histogram data.

The morphology and 3D microstructure of gradient scaffolds were evaluated with SEM and micro-CT analyses. Pore size is one of the most important features of scaffolds to provide an efficient microenvironment for cell proliferation and differentiation. Scaffolds should have a convenient pore size for cell attachment and proliferation as well as oxygen and nutrient transfer for tissue regeneration. In addition, a homogeneous pore size distribution provides a stable microstructure. This homogeneous 3D morphology leads to appropriate mechanical properties for tissue regeneration.^38^ In the literature, the average pore size is given in the range of 200–300 μm for bone cell attachment and proliferation, whereas this range changes to 150–200 μm for cartilage cells.^39^ The pore diameters of laminal and porous layers should be at a sufficient size range that both the cartilage and bone cells can migrate and proliferate at each layer successfully. In this study, the pore size and morphology of CHI-M and CHI-S scaffolds were investigated with SEM analysis (Figure 1). SEM micrographs revealed that the upper layer was obtained as a laminal microstructure, whereas the bottom layer showed a microporous morphology. In addition, a smooth and gradient transient layer was obtained between these two layers. The pore distribution was homogeneous in both porous layers of CHI-M and CHI-S scaffolds. The average pore sizes of each layer were depicted with histogram data (Figure 1). The results showed that the average pore size of laminal structures was in the range of 172–220 μm, whereas the pore size range for bottom layers was between 305 and 318 μm. Each layer showed a favorable microstructure for bone and cartilage layers with appropriate pore size ranges.

**Figure 2:**
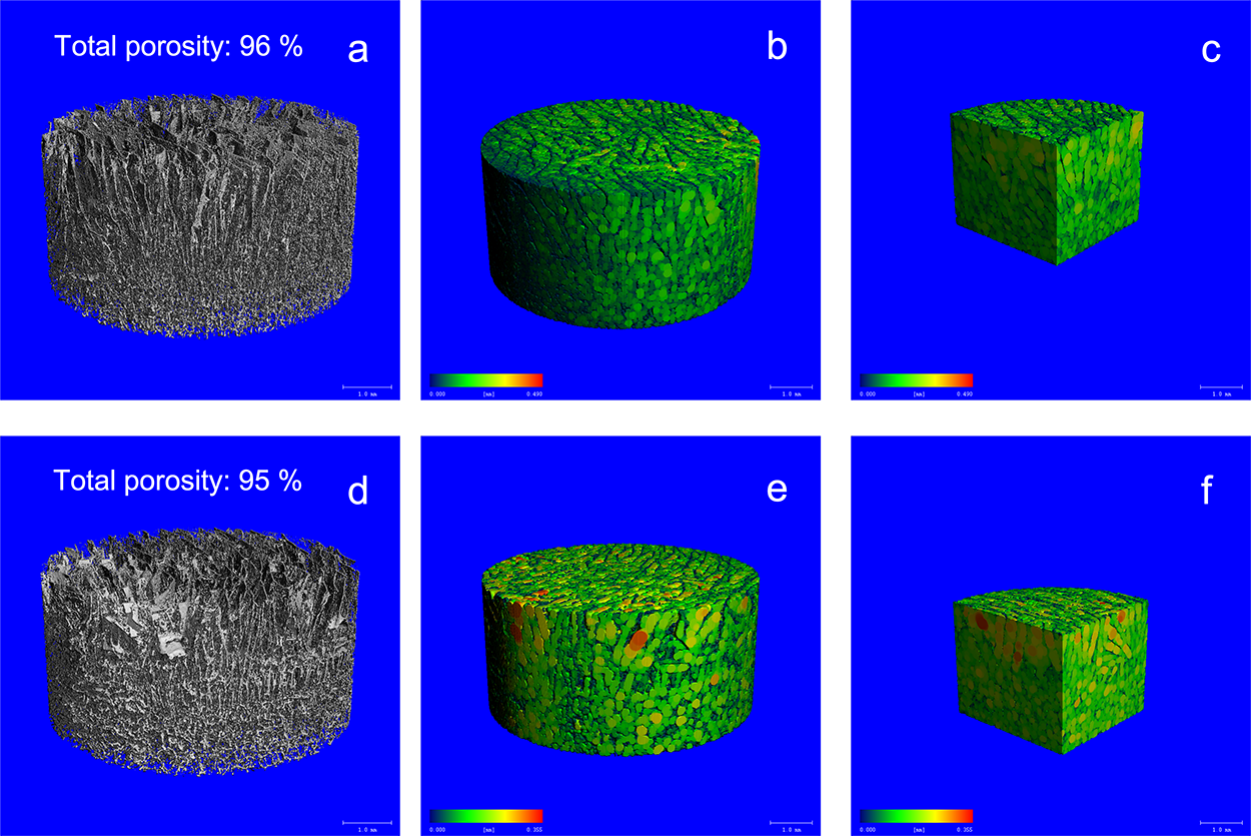
Micro-Ct images of CHI-M (a–c) and CHI-S (d–f) gradient scaffolds: 3D structure, colored pore size distribution, and cross-sectional view, respectively.

Micro-CT analysis revealed that gradient scaffolds were obtained with high total porosity of 96 and 95% for CHI-M and CHI-S, respectively. Micro-CT images indicated that the whole microstructure of gradient scaffolds was obtained with a homogeneous pore distribution (Figure 2b,e). In addition, cross-sectional view images showed the transient layer between upper and lower parts of scaffolds.

### Water Absorption Capacity of Scaffolds

**Figure 3:**
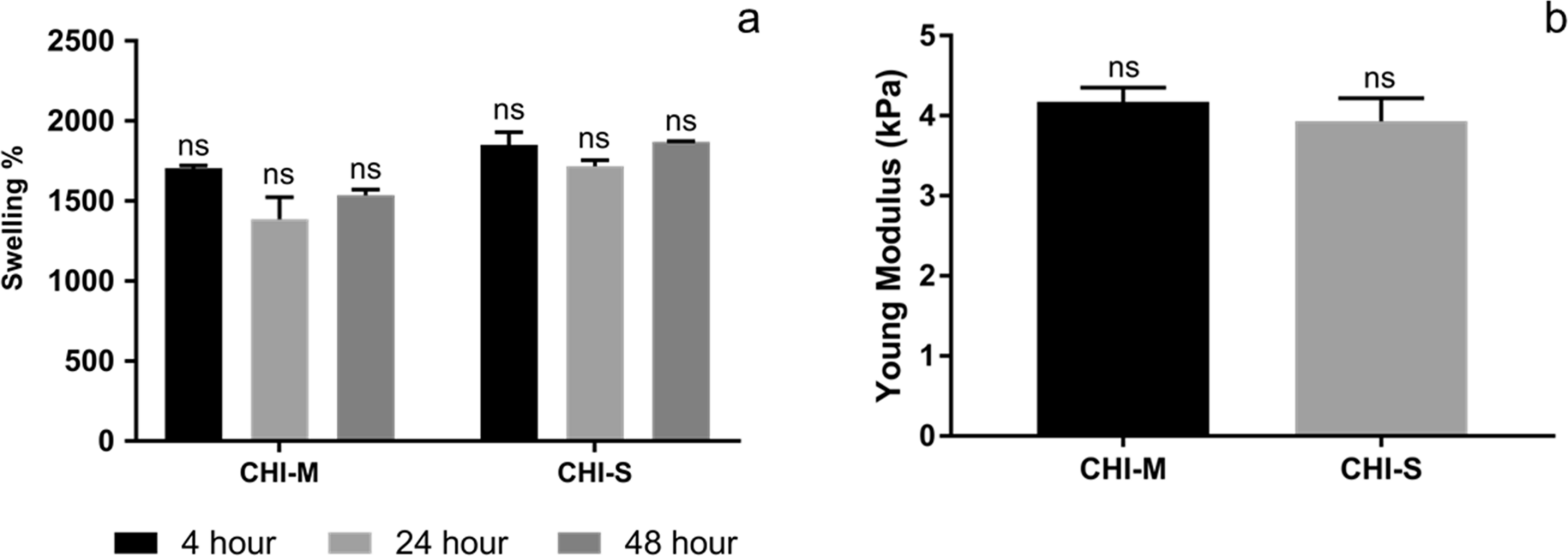
Swelling percentage (a) and Young’s modulus (b) of gradient scaffolds.

The water absorption capacity is a significant factor that affects tissue regeneration. Scaffolds should have a high swelling behavior to provide protein absorption, nutrition, and cell activation for tissue regeneration. The results of CHI-M and CHI-S scaffolds are shown in Figure 3a. The CHI-M and CHI-S gradient scaffolds’ water absorption capabilities were measured at high values of 1704 and 1851% over a 4 h incubation period. At 24 h, the gradient scaffolds’ swelling percentage had barely changed. At 48 h, the water absorption capacity of CHI-M and CHI-S gradient scaffolds increased up to 1535 and 1870%, respectively. No statistically significant difference was detected between groups. These high swelling ratio results arise from the hydrophilic nature of the chitosan matrix and free amino and hydroxyl groups that are distributed throughout the polymer chain. In addition, hydrophilic characteristics of both mucus and slime extracts induce water absorption capacity in a synergistic way.^40−42^ Water absorption capacities of scaffolds were found to be convenient for osteochondral defect regeneration compared with the other studies in the literature.^43−45^

### Mechanical Properties of Scaffolds

The mechanical characteristics of osteochondral tissue differ between the superficial zone and subchondral bone. These unique mechanical properties of osteochondral tissue play a significant role for scaffold design. The upper layer and bottom layer should be mimicking the mechanical strength of both cartilage and subchondral layers. Thus, in this study, mucus and slime extracts were used at a higher concentration (1%) for the bottom layer, and a lower concentration (0.5%) was used in the chitosan matrix for the upper layer to mimic each tissue compartment. The mechanical strength of CHI-M and CHI-S scaffolds was determined by a compression test (Figure 3b). The Young’s modulus of the CHI-M scaffold was 4.2 kPa, whereas that of the CHI-S scaffold was 3.9 kPa. Results indicated that no statistically significant difference was observed between CHI-M and CHI-S gradient scaffolds. In our previous study, mechanical properties of the single-layer MMW chitosan scaffold were investigated. Compression results indicated that the Young’s modulus of the MMW CHI scaffold was 0.8 kPa. The mechanical characteristics of the chitosan matrix were improved by adding mucus and slime extract. The Young’s modulus was raised from 0.8 to 1.76 kPa for 0.5% mucus loading and to 3.07 kPa for 1% mucus loading. The Young’s modulus was likewise raised by slime extract loading, increasing from 0.8 to 2.05 kPa at 0.5% ratio and 3.06 kPa at 1% ratio.^34^

### Exogenous Mineralization on the Scaffold Surface

**Figure 4:**
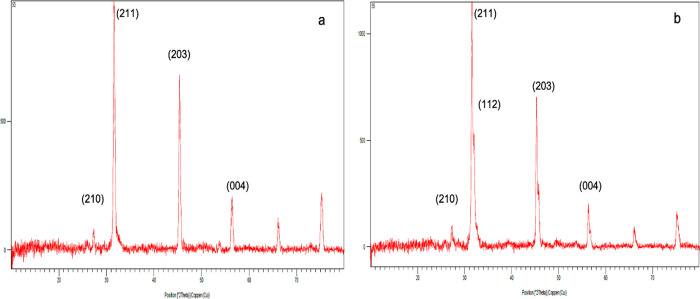
XRD patterns of apatite formation on CHI-M (a) and CHI-S (b) gradient scaffolds immersed in 10× m-SBF at the 14th day.

The biomineralization capacity of CHI-M and CHI-S gradient scaffolds was examined with an exogenous mineralization study using 10× modified SBF solution to mimic the *in vivo* microenvironment in the biomineralization process. Scaffolds were incubated in SBF solution for 14 days. XRD results indicated that characteristic peaks of hydroxyapatite (Hap) crystals were found as (112), (210), (211), (203), and (004) formed on the scaffold surface at the 14th day (Figure 4a,b). The elemental composition of snail secretions mainly consists of Cl, Ca, K, P, Mg, and S, which functionally effects the formation of a growing mollusk shell. In addition, snail mucus contains Si at a trace amount.^46,47^ Thus, the elemental composition of mucus and snail extracts may induce Ca-P formation and Hap mineralization on the chitosan matrix surface.

### *In Vitro* Studies

#### Determination of *In Vitro* Cytotoxicity

**Figure 5:**
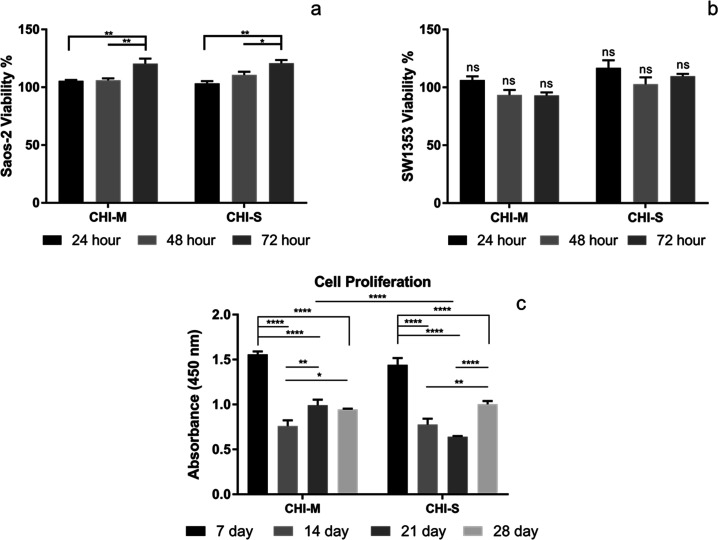
*In vitro* cytotoxicity with Saos-2 (a) and SW1353 (b) cell lines and cell proliferation (c) on CHI-M and CHI-S gradient scaffolds.

The cytotoxicity of CHI-M and CHI-S gradient scaffolds was evaluated with both Saos-2 and SW1353 model cell lines to mimic the bone and cartilage tissue microenvironment. Both Saos-2 and SW1353 cells highly proliferated, and no cytotoxic effect was observed for scaffold extraction media (Figure 5a,b). Results indicated that scaffold extraction media showed a proliferative effect on Saos-2 and Sw1353 cell lines. The cytotoxicity of snail mucus has been studied in the literature. Results indicated that the snail mucus extract induced the cell viability by showing a proliferative effect on fibroblast and epithelial cells.^42,48^ Similarly, in our previous study, we investigated the cytotoxic effect of mucus and slime extract loaded chitosan scaffolds with different concentrations. Single-layer mucus and slime extract loaded scaffolds showed no cytotoxic effect on SW1353 and Saos-2 cell lines.^34^

#### Cell Attachment and Spreading on Gradient Scaffolds

**Figure 6:**
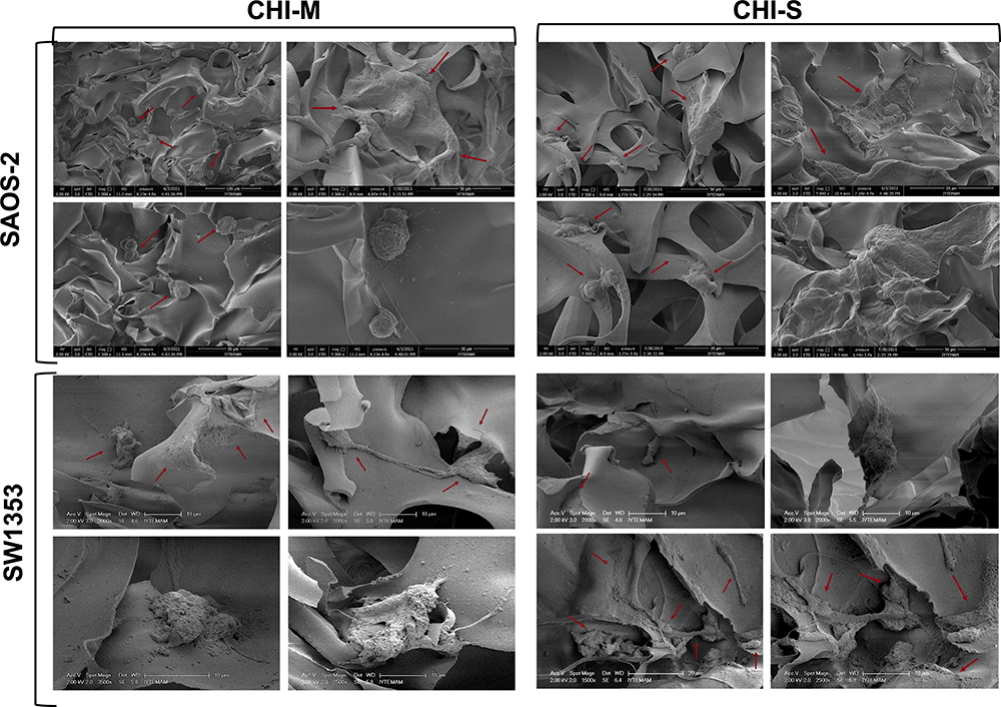
SEM images of Saos-2 and SW1353 attachment and spreading on CHI-M and CHI-S gradient scaffolds at the seventh day of incubation.

SW1353 and Saos-2 attachment and spreading on the upper and bottom layers of gradient scaffolds were examined with SEM analysis at the seventh day of cultivation. SEM micrographs showed that both Saos-2 cells and SW1353 cells attached and spread by colonizing on the pore wall surface of each layer (Figure 6). Saos-2 cells showed a 3D morphology and spread on the surface, whereas SW1353 cells showed an elongated morphology with cell-to-cell interaction on the scaffold surface (red arrows on Figure 6). It is indicated that mucus and slime show distinct characteristics and that mucus has a unique protein content leading to an adhesive characteristic.^49^ Thus, cell spreading on CHI-S scaffolds is significantly observed compared to the CHI-M surface.

#### Cell Proliferation

The cell proliferation on gradient scaffolds was examined by co-culturing Saos-2 and SW1353 cells for 28 days (Figure 5c). The cell proliferation on scaffolds was found to be higher at the seventh day of incubation. Later incubation times, however, resulted in decreased cell population measurements. Instead of cell proliferation, this may be caused by Saos-2 cells’ increased osteoblastic activity and mineralization on mucoadhesive and mineral-containing surfaces.

#### Alkaline Phosphatase (ALP) Activity and Osteocalcin (OC) Secretion of Saos-2 Cells

**Figure 7:**
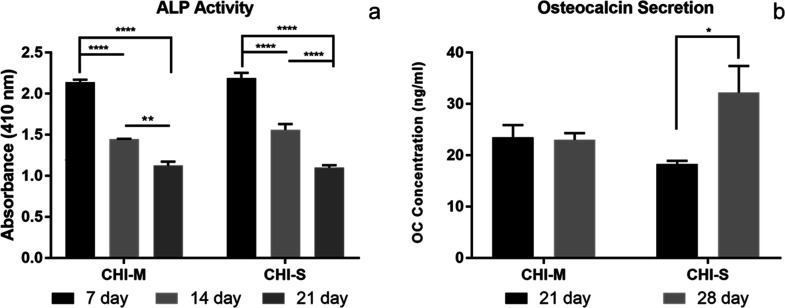
ALP activity and osteocalcin secretion of Saos-2 cells incubated on CHI-M (a) and CHI-S (b) gradient scaffolds.

ALP activity results indicated that Saos-2 cells highly secreted ALP at the seventh day of cultivation on both CHI-M and CHI-S scaffolds (Figure 7a). Results indicated that the ALP secretion level of Saos-2 cells decreased gradually after 7 days of incubation. This may arise from the high secretion levels at the beginning of the cultivation period leading to the initiation of the biomineralization process on the scaffold surface at the early incubation period. Osteocalcin secretion results also proved that the decrease in ALP levels is in good accordance with the biomineralization process initiated at the 21st day (Figure 7b).

#### COMP and GAG Production of SW1353 Cells

**Figure 8:**
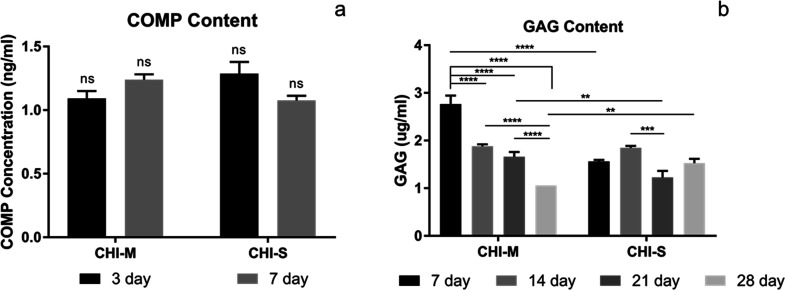
COMP (a) and GAG (b) production of SW1353 cells incubated on CHI-M and CHI-S gradient scaffolds.

The extracellular matrix is mainly composed of GAGs that show important physiological functions such as providing resistance to compressive loading. The cartilage tissue is composed of glycosaminoglycans (15–25%) that are secreted by mature chondrocytes in the extracellular matrix. Chondroitin sulfate (CS), keratan sulfate (KS), and HA are GAGs that form the structure of the articular cartilage.^50−52^ Thus, GAG production is a significant biomarker for cartilage tissue formation. COMP, which is found in the cartilage tissue as a glycoprotein, functions at cell surfaces and the extracellular matrix, playing a part in preserving matrix mechanical properties.^53,54^ Therefore, COMP and GAG production of SW1353 cells was evaluated as significant biomarkers in our *in vitro* studies. Snail secretions have various bioactive components. Allantoin; collagen; elastin; glycolic acid; GAG; and vitamins A, E, and C are among the many components found in the mucus and slime that snails secrete.^32,33^ These components may stimulate the synthesis of COMP and GAG for ECM formation. Figure 8 shows the COMP and GAG secretion of SW1353 cells incubated on CHI-M and CHI-S gradient scaffolds. Results indicated that COMP secretion was statistically similar for mucus and slime loaded scaffolds (Figure 8a). However, COMP secretion showed an increasing trend for the mucus loaded CHI group, whereas a decrease in COMP production was observed for the slime loaded CHI group with the incubation period. The total GAG content of SW1353 cells was determined spectrophotometrically on CHI-M and CHI-S scaffolds during 28 days of incubation (Figure 8b). Results indicated that the highest GAG content was obtained for CHI-M groups at the seventh day.

#### Determination of Extracellular Matrix Component GAG with Alcian Blue Staining

**Figure 9:**
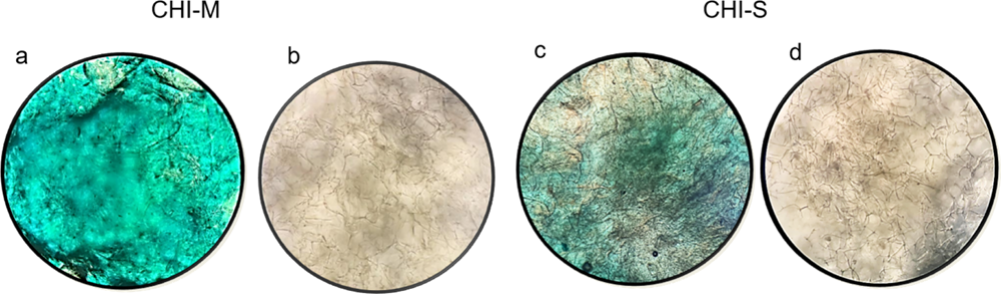
Stereoimages of gradient scaffolds stained with Alcian Blue (a, c) and von Kossa (b, d) for ECM production and biomineralization, respectively.

SW1353 cells incubated on the upper layer of gradient scaffolds were fixed at the end of the incubation period (28 day). Then, scaffolds were stained with Alcian Blue to detect the GAG production on the material surface (Figure 9a,c). Light microscopy images indicated that SW1353 cells secreted GAG on the laminal microstructure of both mucus and slime loaded chitosan scaffolds. However, microscopy images showed that the mucus extract loaded scaffold induced GAG production significantly compared to the slime loaded group.

#### Biomineralization with von Kossa Staining

Stereoimages of von Kossa-stained scaffolds are depicted as Figure 9b,d. Images show the phosphate deposition bound to calcium minerals on the CHI-M and CHI-S scaffold surface at the 28th day of incubation. Therefore, von Kossa staining indicates the calcium-phosphate formation potential of scaffolds indirectly. Calcium deposition was obtained for all scaffolds owing to the calcium carbonate granule content of *H. aspersa* secretions.^55^ Stereoimages indicated that phosphate deposition was slightly seen on the mucus loaded scaffold surface; however, slime incorporation enhanced the biomineralization at the center of the scaffold surface as the heterogeneous brown color changing by accumulating phosphate deposition.

### Histological and Immunohistochemical Staining

#### Histological Staining

**Figure 10:**
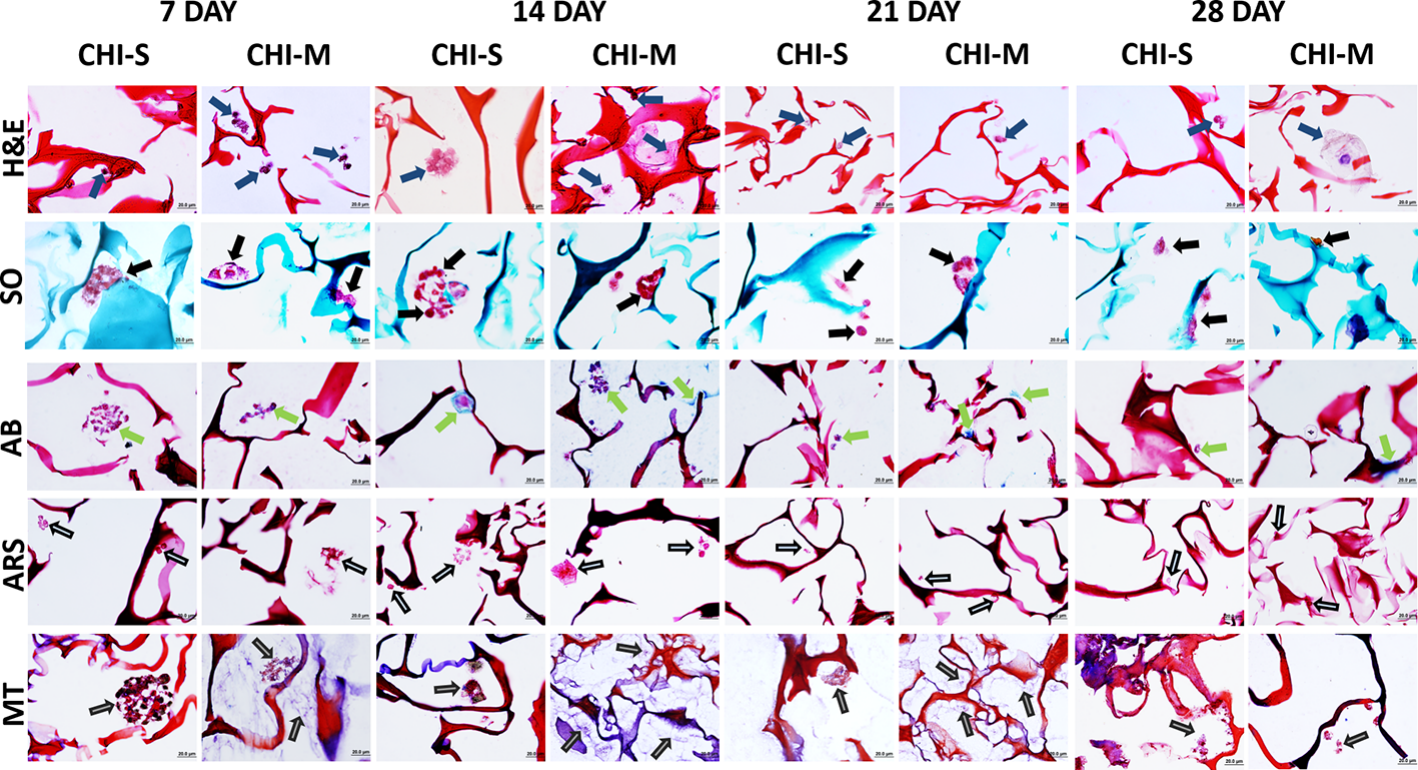
Hematoxylin and eosin (H&E), Safranin O (SO), Alcian Blue (AB), Alizarin Red S (ARS), and Masson’s Trichrome (MT) staining images of gradient scaffolds at 7, 14, 21, and 28 days of incubation.

Histochemical evaluations of extracellular matrix (ECM) components expressed by osteogenic (Saos-2) and chondrogenic (SW1353) carcinoma cells in gradient scaffolds were performed at 7, 14, 21, and 28 days (Figure 10). Pericellular matrix deposition was observed further in the CHI-S scaffold at the 14th day. In addition, collagen deposition and GAG uptake increased on the 14th day in the CHI-M scaffold compared to other groups. In Alizarin Red S staining, calcium depositions were found to be similar in both scaffolds on the 7th and 14th days and decreased on the other days. H&E staining was used to observe the general morphology of the cells, where Safranin O/Fast Green (red/purple) and Alcian Blue (green/light blue) stainings were used for proteoglycan and GAG detection, respectively. Alizarin Red S (red/purple) and Masson’s Trichrome (blue) stainings were used for the detection of inorganic calcium and collagen, respectively. On the 14th day, it was determined that CHI-S and CHI-M scaffolds provided cell proliferation and migration with the help of adhesion of the specific extracellular matrix components having both osteogenic and chondrogenic properties. By this way, the cell adhesion on the material surface was enhanced. It was also found that the biological effect of the CHI-S scaffold showed better *in vitro* biological activity than the CHI-M scaffold. On the 7th and 14th days, both CHI-M and CHI-S scaffolds showed a homogeneous cell distribution on the scaffold surface, and spheroid-shaped cell clusters were observed at the tip of the pores (Figure 10). However, cells were distributed one by one on the 21st and 28th days on the material surface. In addition, it was determined that cell proliferation, migration, and metabolic activities decreased compared to those at the 7th and 14th days. For both cell types, GAG and collagen formation increased on all scaffold groups (especially CHI-M) on day 14 but decreased on the 21st and 28th days (Figure 10). Calcium deposition on CHI-S and CHI-M scaffolds was found to be similar at the 7th and 14th days, and a significant decrease was observed in the following days (Figure 10). The increasing trend of the extracellular matrix synthesized on CHI-M scaffolds supports that the biochemical properties of mucus incorporation may have an important role in modulating osteocyte and chondrocyte function. It was observed that the slime loaded scaffolds had a greater inducing effect on cell morphology, proliferation, and migration.

#### Immunohistochemical Staining

**Figure 11:**
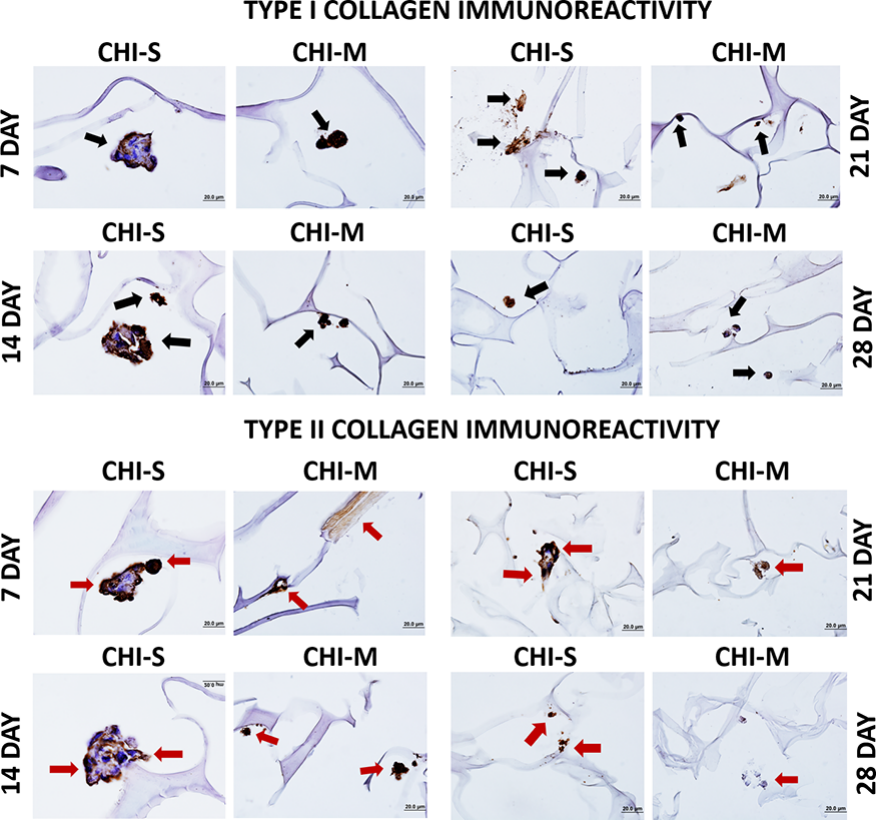
Immunohistochemical staining images of gradient scaffolds at the 21st and 28th days showing type I and type II collagen production: black arrows indicate type I collagen positive cells, and red arrows indicate type II collagen positive cells.

Type I collagen and type II collagen immunoreactivity expressed by osteogenic (Saos-2) and chondrogenic (SW1353) carcinoma cells in gradient scaffolds is depicted in Figure 11. Type I collagen and Type II collagen positive cell immunoreactivity was observed in CHI-S and CHI-M scaffolds on 4 different days. At the 14th day of incubation, type I collagen reaction intensity increased in the CHI-S scaffold, whereas the intensity of the type II collagen reaction increased in the CHI-M scaffold. In the CHI-S scaffold group, type I collagen fibrils accumulated in the pericellular area at the seventh day, whereas an uptake was observed in the entire spheroid-shaped cell aggregation at the 14th day (Figure 11). Type II collagen positive cell immunoreactivity was detected similarly in the pericellular area of the CHI-S scaffold on days 7 and 14. At the 14th day, an increase in the reaction intensity of type II collagen was observed for the CHI-M scaffold compared to the 7th day. The reaction intensity of type I collagen showed a decrease at the 21st and 28th day for both CHI-M and CHI-S scaffolds. The decrease in the reaction intensity of type II collagen was found to be higher than that of type I collagen intensity at the 21st and 28th days (Figure 11).

## Conclusions

In this study, CHI-M and CHI-S scaffolds were successfully fabricated via the layer-by-layer freezing and freeze-drying technique to obtain the gradient form as a continuous 3D structure. Characterization results indicated that both scaffolds showed highly porous morphology, good swelling behavior, and mechanical properties for osteochondral tissue. XRD results indicated that both mucus and slime extract incorporation induced exogenous mineralization on the scaffold surface. Water absorption capacities of CHI-M and CHI-S scaffolds were found to be convenient for osteochondral defects. SW1353 and Saos-2 cells were co-cultured on each layer of gradient scaffolds. *In vitro* bioactivity results indicated that slime extract incorporation significantly induced biomineralization by increasing osteocalcin secretion as well as phosphate deposition in von Kossa staining images. Both spectrophotometric GAG analysis and Alcian Blue staining results indicated that mucus extract loading increased the GAG content compared to slime extract loaded groups. Histological and immunohistochemical analyses of scaffolds also supported *in vitro* bioactivity results. In conclusion, it was determined that our novel gradient scaffold design was a promising one for simulating the osteochondral region. Additionally, highly bioactive mucus and slime extracts significantly increased the synthesis of ECM components for tissue regeneration and biomineralization in osteoblast and cartilage model cell lines.
